# Appointments to Insane Hospitals

**Published:** 1860-11

**Authors:** 


APPOINTMENTS TO INSANE HOSPITALS.—Dr. J. P. Clement has been appointed Superintendent of the State Hospital for the Insane, at Madison, Wisconsin, and Dr. R. J. Patterson has been called to fill the same position in the State Hospital for the Insane, at Mount Pleasant, Iowa.

